# Effects of Brown Seaweed (*Sargassum polycystum*) Extracts on Kidney, Liver, and Pancreas of Type 2 Diabetic Rat Model

**DOI:** 10.1155/2014/379407

**Published:** 2014-01-08

**Authors:** Mahsa Motshakeri, Mahdi Ebrahimi, Yong Meng Goh, Hemn Hassan Othman, Mohd Hair-Bejo, Suhaila Mohamed

**Affiliations:** ^1^Faculty of Food Science & Technology, Universiti Putra Malaysia, 43400 Serdang, Selangor, Malaysia; ^2^Faculty of Veterinary Medicine, Universiti Putra Malaysia, 43400 Serdang, Selangor, Malaysia; ^3^Institute of Tropical Agriculture, Universiti Putra Malaysia, 43400 Serdang, Selangor, Malaysia; ^4^Institute of Bioscience, Universiti Putra Malaysia, 43400 Serdang, Selangor, Malaysia

## Abstract

The edible seaweed *Sargassum polycystum* (SP) is traditionally used against several human diseases. This investigation evaluated the effects of two dietary doses of SP ethanolic and aqueous extracts on the pancreatic, hepatic, and renal morphology of type 2 diabetic rats (T2DM). T2DM was induced by feeding rats on high calorie diet followed by a low dose streptozotocin. Changes in the diabetic rat organs in SP treated groups with different doses of extracts were compared with normal rats, diabetic control rats, and metformin treated rats. After 22 days of treatment, the pathological lesions of the livers and kidneys in the diabetic rats were quantitatively and qualitatively alleviated (*P* < 0.05) by both the SP extracts at 150 mg/kg body weight and by metformin. All the treated diabetic groups revealed marked improvement in the histopathology of the pancreas compared with the control diabetic group. Oral administration of 300 mg/kg body weight of aqueous and ethanolic extracts of SP and metformin revealed pancreas protective or restorative effects. The seaweed extracts at 150 mg/kg body weight reduced the liver and kidney damages in the diabetic rats and may exert tissue repair or restoration of the pancreatic islets in experimentally induced diabetes to produce the beneficial homeostatic effects.

## 1. Introduction

Diabetes mellitus is an endocrine disorder characterized by defects in carbohydrate, lipid, and protein metabolism. It is a leading cause of morbidity and mortality worldwide, due to diabetic complications such as coronary heart disease, stroke, retinopathy, nephropathy, liver disease, and peripheral neuropathy [1]. The majority (about 90%) of diabetes is of Type 2 (T2DM) or non-insulin-dependent diabetes mellitus (NIDDM), which is the result of deviations in pancreatic *β*-cells functions, insulin secretions, and insulin insensitivity [2]. Hyperglycemia increases the production of free radicals [3], and induces oxidative stress leading to liver injuries related to carbohydrate metabolism disorder [4, 5]. These injuries are represented by cellular degenerations, pyknotic nuclei, and cellular necrosis due to increased lipid accumulation and oxidation in the hepatocytes. However, the liver is able to regenerate even after initial injuries [6]. Various diabetic complications are caused by defects in the body antioxidant defence systems [7], oxidative stress, and damages to cellular membranes, subcellular organelles [8–11], DNA damage, and cell death [12].

Natural antioxidants from plants retard these damages, and may be an effective, safe, and economical alternative therapy for diabetes management and organs protection. *In vivo* studies and histopathological examinations are necessary to prove their efficacy and safety on the liver, kidney, pancreas, and the other important organs, since biochemical measurements alone are not conclusive. The common edible brown seaweed *Sargassum polycystum *(*C. Agardh*) (SP) reportedly alleviated hyperglycaemia and dislipidemia in diabetic rats [13], possibly due to its good antioxidant and free radical scavenging properties [14]. SP is reportedly used for eczema, scabies, and psoriasis, ulcer and lung diseases, renal dysfunction, viral hepatitis and heart ailments and to promote bile secretion [7], besides having antilipidemic, antioxidant and membrane stabilizing properties [8, 15]. SP also has drug metabolizing enzymes protective effects, prevents TNF-*α* elevation [9], inhibits lipid peroxidation, and preserves hepatic antioxidant defence system *in vivo* [10, 12]. It was reported to be hepatoprotective under high-fat/high-cholesterol diet [16]. The administration of SP ethanolic or water extracts dose dependently reduced blood glucose, glycosylated hemoglobin (HbA1C) levels, and dyslipidemia in type 2 diabetic animals [13]. SP appeared to be an insulin sensitizer, beneficial in the management of T2DM that can also help reduce atherogenic risk [13]. Currently, there is no report the organ protective effect of SP in type 2 diabetes animal model. This study reports on the protective or tissue restorative effects of SP ethanolic and water extracts on the pancreas, liver, and kidney tissues in type 2-induced diabetic rat model.

## 2. Materials and Methods

### 2.1. Seaweed Material

SP was collected from the northeast coast of Borneo (Semporna, Sabah, Malaysia) and was identified by Dr. P. Matanjun, University Malaysia Sabah. A voucher specimen (PSP5) of the seaweed was preserved in the Borneo Marine Research Institute Herbarium.

#### 2.1.1. Aqueous and Ethanolic Extracts Preparation

The fresh seaweed fronds were washed thoroughly in seawater and then in tap water to remove holdfasts, epiphytes, and sands. Seaweed samples were dried in a 40°C oven and milled to a powder. Seaweed powder (250 g) was extracted with 2.5 L of absolute ethanol (HmbG Chemicals, Germany) at room temperature with occasional shaking for a period of 72 h. The crude extract was filtered, concentrated at 40°C using a rotary vacuum evaporator (Eyela, Tokyo, Japan), and dried in an oven at 40°C for 4-5 h (yield 9.5% on a dry-weight basis). An aqueous extract (yield 6-7% on a dry-weight basis) was obtained by boiling seaweed powder (250 g) with distilled water for up to 12 h. After every 4 h, the solution was decanted and the residue was reextracted with new distilled water (4 L). The residue was then strained through cheese cloth and all extracts were centrifuged at 4000 rpm for 20 min to remove particulate substances. Eventually the supernatant was freeze-dried under reduced pressure (2 mmHg) at −20°C (FDU-1200, Eyela, Tokyo, Japan). The resulting dried powder was used in the experiment.

### 2.2. Animal Models

Male *Sprague-Dawley* rats, weighing approximately 200–250 g, were obtained from a local supplier (Saphire Enterprise Sdn. Bhd, Serdang, Malaysia). Animals were acclimatized for 1 week in individual cages, at 23 ± 2°C with 60–75% relative humidity and a 12 h light/12 h dark cycle, with free access to standard rat diet (Gold Coin Co., Klang, Malaysia) and water. All procedures used were in accordance with the guidelines on the ethical use and care of laboratory animals issued by the Faculty of Veterinary Medicine, University Putra, Malaysia (Approval number UPM/FPV/PS/IAUC no. 3.2.1.551/AUP-R18).

#### 2.2.1. Induction of Type 2 Diabetes in Rats

Type 2 diabetes was induced by feeding on high-sugar high-fat diet (HSHFD) for 16-weeks followed by a single intraperitoneal injection of freshly prepared streptozotocin (35 mg/kg BW STZ; Sigma) dissolved in sterile saline solution (9 g/kg NaCl) [13]. The rats on the normal control group received an equivalent volume of saline solution. The fasting blood glucose levels were checked 48 h after injection using the glucometer (ACCU-Check, Roche Diagnostics Corporation, USA). The animals were considered diabetic with fasting blood glucose values greater than 11 mmol/L [17]. The high-sugar high-fat diet (HSHFD, 4.29 kcal/g) was prepared by mixing 15% w/w of plant-based margarine (Planta; Unilever Corporation Sdn. Bhd., Kuala Lumpur, Malaysia) with standard rat diet (3.77 kcal/g), accompanied by 30% refined sugar (CSR Corporation Sdn. Bhd., Selangor, Malaysia); solution was provided as the drinking fluid. These were prepared daily and unconsumed food over 24 h was discarded in order to avoid oxidation and rancidity. Animals considered as normal control received standard rat diet with *ad libitum* distilled water till the end of the experiment.

#### 2.2.2. Animals Experimental Design

Forty-two rats were randomly divided into seven groups (*n* = 6) as follows: Group (a): normal control rats (NC; untreated and nondiabetic), Group (b): untreated diabetic control (DC), Group (c): diabetic rats treated with 150 mg ethanolic extract kg^−1^ body weight (DE150), Group (d): diabetic rats treated with 300 mg ethanolic extract kg^−1^ body weight (DE300), Group (e): diabetic rats treated with 150 mg water extract kg^−1^ body weight (DW150), Group (f): diabetic rats treated with 300 mg water extract kg^−1^ body weight (DW300), Group (g): diabetic rats treated with 250 mg metformin hydrochloride kg^−1^ body weight. The metformin tablets (Hovid Corporation Sdn. Bhd., Kuala Lumpur, Malaysia) were crushed and dissolved in distilled water was used as the reference drug or positive control (DM). The extracts and metformin were administered once daily by oral gavage on overnight fasted rats for 22 days after diabetes induction. The 150 mg and 300 mg kg^−1^ SP doses were within the dose range for metformin and equivalent to about 50–100 g of seaweed kg^−1^ diet.

#### 2.2.3. Blood Glucose Monitoring

Blood glucose was monitored at 1, 7, 13, 19, and 22 days after-treatments of diabetic and normal rats according to [18]. Blood was collected from the tail of the animals after 12–16 h overnight fasting. The tail tip was sanitized with alcohol and pricked and a drop of blood was used for blood glucose measurement using a glucometer (ACCU-Check, Roche Diagnostics Corporation, USA).

#### 2.2.4. Tissue Collection and Histopathology

At the end of the experiment, the pancreas, liver, and kidney tissues were promptly excised from the sacrificed animals according to [19, 20]. The organs were rinsed with normal saline and fixed into 10% neutral buffered formalin for histopathological examinations. The fixed tissues were then cut into small sizes and were put in a labelled tissue cassette for dehydration processing (LEICA, ASP 300, Nussloch, Germany). After dehydration with a series of different ethanol concentrations (70%, 95%, and 100% (5 times), resp.), the tissues were cleaned twice with xylene before being embedded in paraffin molds (LEICA, EG 1160, Nussloch, Germany). Each cooled paraffin block was sliced to 4 *μ*m thicknesses using a microtome (LEICA, RM2155, Nussloch, Germany). Each section was floated on a 45°C water bath and picked up using a glass microscope slide (LEICA, HI 1210, Nussloch, Germany). The sections were then dried at 58°C on a heater (LEICA, HI 1220, Nussloch, Germany) for 15 minutes to melt the wax and to secure the tissue firmly on the glass slide. For the hematoxylin and eosin (H and E) staining, the sections were routinely deparaffinised using xylene and rehydrated through a series of descending alcohol concentration and water mixtures (100%, 90%, and 70%). Hematoxylin (H) stains the cell nuclei and Eosin (E) stains the cytoplasmic components. Slides were then passed through series of alcohol for dehydration (70%, 90%, and 100%, resp.), cleared by xylene, mounted with cover slip, and examined under light microscope (Olympus BX51, Tokyo, Japan).

#### 2.2.5. Lesion Scoring for Pancreas, Liver, and Kidney

Morphological changes related to degenerated and necrotic cells were counted in five fields of each tissue section of endocrine pancreas, liver, and kidney using an image analyzer (Olympus BX51, Tokyo, Japan) at 200x magnifications. The degree of injuries was expressed as the percentage mean of lesions in five different fields (zigzag manner) in each section [21].

### 2.3. Statistical Analysis

Normalized data of the percentage of lesions in the organs were analyzed as a completely randomized design experiment using the General Linear Model (GLM) of SAS (Statistical Analysis Systems Institute Inc., 1992). The LSD test was used to differentiate the means. All data were expressed as mean ± SE (standard error) and *P* < 0.05 was identified as significantly different.

## 3. Results

### 3.1. Effect of Seaweed Ethanolic and Aqueous Extracts on the Blood Glucose

After induction of diabetes by HSHFD + low dose STZ, diabetes was confirmed by the presence of hyperglycemia, polyuria, and polydipsia in the animals. The levels of blood glucose in (HSHFD + STZ)-induced diabetic rats were significantly (*P* < 0.05) elevated as compared with normal control rats (Table 1). Oral administration of ethanolic and aqueous extract of SP (150 and 300 mg/kg body weight) to diabetic rats for 22 days caused significant reduction in blood glucose levels (Table 1). The hypoglycemic activity of the extracts was of the order DM > DE300 > DW300 > DE150 > DW150 in diabetic rats.

### 3.2. The Effects of SP Extracts on the Pancreas Tissue Morphology

**Figure 1:**
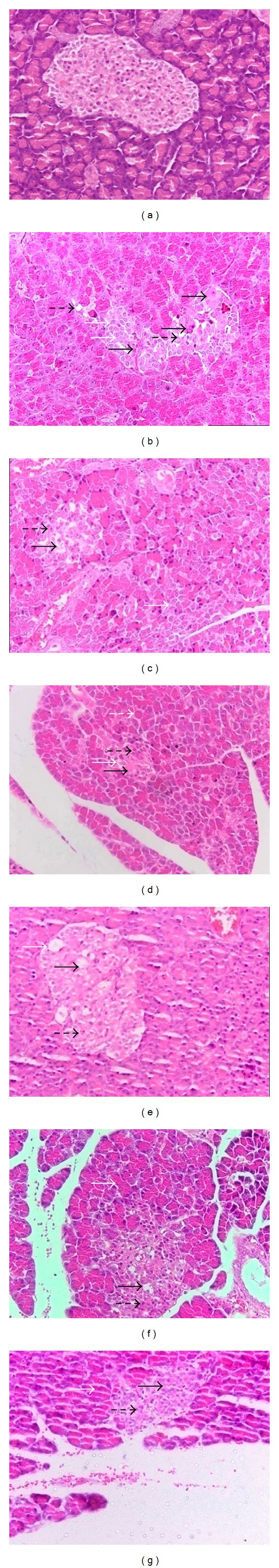
Photomicrographs of pancreas tissues of rats from different experimental groups. (a) Normal control rats pancreatic endocrine and exocrine showing islets of Langerhans and acini (200x). (b) Diabetic rats pancreas shows degeneration and shrinkage (white arrow), vacuolar change (dashed black arrow), necrotic cells with pyknotic nuclei and acidophilic cytoplasm (black arrow), and disruption of normal endocrine architecture (200x). (c) Pancreas of metformin treated diabetic rats shows degeneration and shrinkage (irregular space in the islets of Langerhans—black arrow), swelling of the acinar epithelial lining and irregular arrangements (luminal disappearing—white arrow), and reduced necrosis (dashed black arrow) (200x). (d) Pancreas of 300 mg ethanol extract treated diabetic rats shows significantly reduced necrosis (dashed black arrow), reduced degeneration (black arrows), reduced irregular spaces (white arrow), and normal acinar epithelial lining (dashed white arrow) (200x). (e) Pancreas of 300 mg water extract treated diabetic rats shows significant cellular and architectural restoration, reduced necrosis (dashed black arrow), reduced degeneration (black arrows), minimum vacuolar degeneration (white arrow), and normal endocrine and exocrine (dashed white arrow) (200x). (f) Pancreas of 150 mg ethanol extract treated diabetic rats shows multifocal area of necrosis condensed nuclei and acidophilic cytoplasm (dashed black arrow), reduced cellular degeneration and reduced vacuolar swelling (black arrows), and normal exocrine (white arrow) (200x). (g) Pancreas of 150 mg water extract treated diabetic rats shows slight necrosis reduction (dashed black arrow), cellular degeneration and acute swelling (absence of spaces in the islets of Langerhans—black arrows), and irregular arrangement of the acinar epithelial lining (white arrow) (200x).

The photomicrograph of the pancreas in normal control rats showed characteristic features of normal acini and normal cellular population in the islets of Langerhans (Figure 1(a)). In contrast, the DC rats demonstrated atrophy and severe injuries represented by pyknotic nuclei and acidophilic cytoplasm in the necrotic cells along with vacuolar changes in degenerative cells (Figure 1(b)). The severity of these injuries was alleviated markedly (*P* < 0.05) in all diabetic rats treated with either the extract or metformin (Figures 1(c)–1(g), Table 2). The extent of necrotic cells decreased significantly (*P* < 0.05) in the treated groups with the order of DW300 < DE150 < DE300 < DW150 < DM; however, in terms of total damages all showed moderate level of cell damages as compared with the DC and NC groups (Table 2). Interestingly, cellular component of the islets of Langerhans in DM (Figure 1(c)), DE300 (Figure 1(d)), and DW300 (Figure 1(e)) groups showed some restoration compared to the other groups. Remarkably, the percentage of degenerative cells in islets of DM group was significantly (*P* < 0.05) lower compared to the seaweed treated groups (Table 2).

### 3.3. Effects of SP Extracts on the Hepatic Tissue Morphology

**Figure 2:**
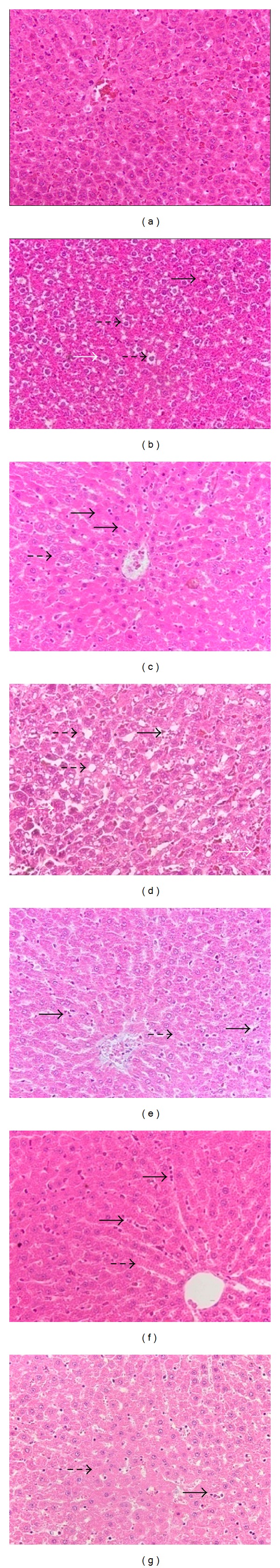
Photomicrographs of liver tissues of rats from different experimental groups. (a) Hepatocytes of normal control rats shows insignificant cellular swelling and sinusoidal congestion (200x). (b) Diabetic rats liver show severe degeneration and fatty changes (white arrow), glycogen deposition (dashed black arrow), necrotic cells with pyknotic nuclei and acidophilic cytoplasm (black arrow), and cellular swelling (200x). (c) Liver of metformin treated diabetic rats shows mild granular degeneration (dashed black arrow) and mild swelling (narrow sinusoidal capillaries—black arrow) and normal hepatic architecture (200x). (d) Liver of 300 mg ethanol extract treated diabetic rats shows significantly reduced fatty change (dashed black arrow), multifocal infiltration of inflammatory cells (black arrows), sinusoidal congestion (white arrow), and granular degeneration (dashed white arrow) (200x). (e) Liver of 300 mg water extract treated diabetic rats shows normal morphological architecture and significant degenerative cells reduction (dashed black arrow) and mild to moderate Kupffer cells hyperplasia (black arrows) (200x). (f) Liver of 150 mg ethanol extract treated diabetic rats shows normal architecture and hepatocytes, significantly reduced degenerative cells (dashed black arrow), and mild Kupffer cells hyperplasia (black arrows) (200x). (g) Liver of 150 mg water extract treated diabetic rats shows normal architecture and hepatocytes, significantly missing degenerative cells, mild cellular swelling (narrow sinusoidal capillaries—dashed black arrow), and mild Kupffer cells hyperplasia (black arrows) (200x).

Light microscopic observation of the liver sections of normal control rats showed characteristic features of radiating hepatic cells around a normal central vein with narrow sinusoid, with no significant (*P* > 0.05) sinusoidal congestion and mild cellular swelling (Figure 2(a)). On the contrary, in diabetic rats destructive changes were more evident; the DC rats exhibited nonradiating sinusoids, mild scattered necrotic cells with pyknotic nuclei, and severe degenerations in the hepatocytes such as microvesicular and macrovesicular vacuolation of the hepatocyte cytoplasm, glycogen deposition, fatty changes, and cellular swelling (Figure 2(b)). Treatment with the SP extracts (Figures 2(f)–2(g)) and metformin (Figure 2(c)) showed improvement in histological structure of the liver sections of the diabetic rats with normalized appearance of the liver in the DE150 and mild degree of injuries in the DW150 and the metformin treated animals. In the DM group, the hepatocytes exhibited some degree of histological restorations defined by granular degenerations, and mild cellular swelling. In the DE300 group, the severity of the total injuries was almost similar to the DC group, with even higher percentage of necrotic cells (Table 2). Severe fatty degeneration of the hepatocytes, inflammation, and sinusoidal congestion were also apparent in the liver of the DE300 group (Figure 2(d)). In contrast, the DW300 (Figure 2(e)) and the DW150 (Figure 2(g)) groups displayed significant reduction and missing of degenerative cells altogether. Figures 2(e) and 2(g) showed normal morphological arrangement of the hepatocytes, mild Kupffer cells hyperplasia, macrovesicular vacuoles, and little lipid droplets within the liver.

### 3.4. Effects of SP Extracts on the Renal Tissue Morphology

**Figure 3:**
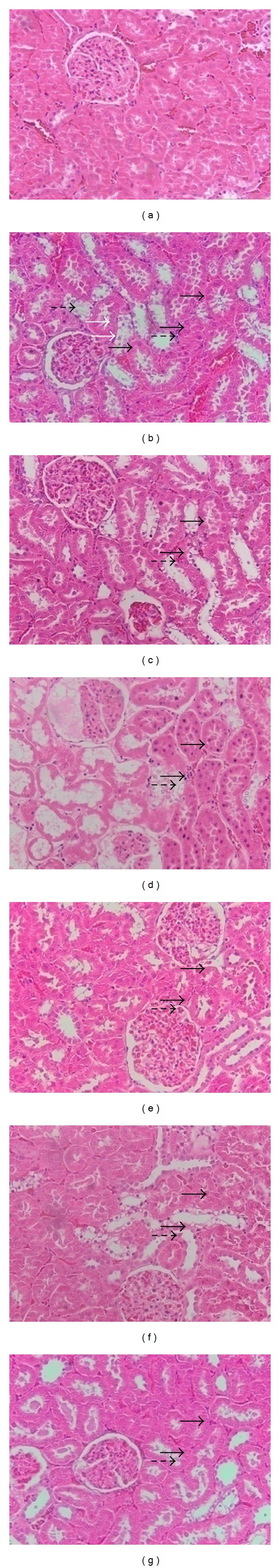
Photomicrographs of Renal tissues of rats from different experimental groups. (a) Renal Cortex of normal control rats show renal corpuscle and tubules with moderate congestion of the cortical blood vessels (200x). (b) Diabetic rats Renal Cortex shows severe cellular injury, hydropic degeneration and swelling (black arrow), glomerular atrophy and widening of the bowman's space (dashed black arrow), and necrotic tubular epithelium (pyknotic nuclei and acidophilic cytoplasm—white arrow) (200x). (c) Renal Cortex of metformin treated diabetic rats shows moderate cellular hydropic degeneration (dashed black arrow), atrophied renal corpuscle (black arrow), and mild glomerular capillaries congestion (white arrow) (200x). (d) Renal Cortex of 300 mg ethanol extract treated diabetic rats shows severe glomerular atrophy and accumulation of protein acious inflammatory pinkish fluid in the glomerular spaces (black arrow), severe necrosis of the tubular epithelium with degenerative cells (dashed black arrows), congestion, and a few inflammatory cells (white arrow) (200x). (e) Renal Cortex of 300 mg water extract treated diabetic rats shows mild cellular degeneration and swelling (star shaped tubular lumen—dashed black arrow), mild fatty changes in the glomerular epithelial capillaries (black arrows), and mild changes in the glomerulus (white arrow) (200x). (f) Renal Cortex of 150 mg ethanol extract treated diabetic rats shows swelling in the tubular epithelium (black arrow), mild congestion, and degeneration of the glomerular capillary cells (dashed black arrow) (200x). (g) Renal Cortex of 150 mg water extract treated diabetic rats shows mild to moderate cellular degeneration (dashed black arrow), mild glomerular atrophy (black arrow), and hyaline cast pinkish amorphous protein within the tubular lumen (white arrow) (200x).

The effects of SP extracts on the diabetic rats' kidneys are shown in Figures 3(a)–3(g). The normal control rats demonstrated normal architecture of the renal corpuscle and renal tubules (Figure 3(a)). In contrast, the kidneys of untreated diabetic rats revealed acute cellular swelling, severe tubular hydropic degenerations, glomerular shrinkage, widening of bowman's space, and severe tubular epithelium necrosis (Figure 3(b)). All the necrotic changes observed in the tubules together with the cellular degenerations in the glomerulus and tubules were found to be alleviated significantly (*P* < 0.05) in the diabetic rats of the DE150, DM, and DW150 groups (Table 2). The percentage number of necrotic cells in kidney sections of DE300 rats significantly (*P* < 0.05) increased as compared with that of DC group, but the percentage level of degenerative cells was significantly reduced (*P* < 0.05) in all the treated diabetic rats (Table 2). Severe glomerular atrophy along with accumulation of proteinaceous inflammatory pinkish fluid in the glomerular space, severe necrotic area in the tubular epithelium together with scattered degenerative cells, infiltration of some inflammatory cells, and blood congestion were observed in the DE300 group (Figure 3(d)). The kidney sections of the DW300 rats displayed mild tubular degeneration, mild cellular swelling, and mild fatty degeneration in the glomerular endothelial capillaries (Figure 3(e)). Quantitatively, cell scoring of DW300 kidney sections showed lower (*P* < 0.05) percentage levels of degenerative and necrotic cells as compared to the DE300 and DC rats (Table 2). The DW150 rats showed a significantly lower (*P* < 0.05) percentage of damages as compared to the DC animals (Figure 3(g)) with mild glomerular atrophy and the presence of hyaline cast, pinkish amorphous protein material within the tubular lumen. Similarly, the kidney sections of the DE150 rats showed mild tubular degeneration and cellular swelling along with mild glomerular capillary cells degeneration and congestion (Figure 3(f)). Similar to the DE150 and DW150 groups, metformin treated rats showed significantly lower (*P* < 0.05) damages as compared to DC rats represented by mild to moderate tubular hydropic degeneration, mild glomerular capillaries congestion, and atrophied renal corpuscle (Figure 3(c)).

**Figure 4:**
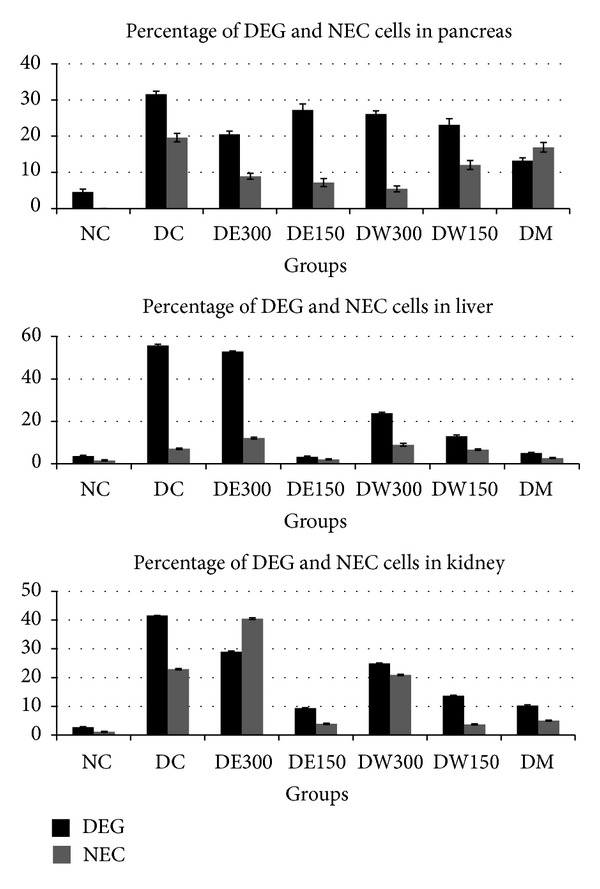
Percentage distribution of degenerated (DEG) and necrotic (NEC) cells in the pancreas, liver, and kidney of rats from different experimental groups. NC: normal control group. DC: diabetic control group. DE300: diabetic group treated with 300 mg/kg/BW of the ethanolic extract. DE150: diabetic group treated with 150 mg/kg/BW of the ethanolic extract. DW300: diabetic group treated with 300 mg/kg/BW of the water extract. DW150: diabetic group treated with 150 mg/kg/BW of the water extract. DM: diabetic group treated with 250 mg/kg/BW of metformin.

The quantitative and qualitative scores (necrosis or degenerating cells) of the injured cells in the pancreas, liver, and kidneys are summarized in Figure 4.

## 4. Discussion

The long-term high calorie diet together with mild pancreatic damage used here provides a new *Sprague-Dawley* rats model suitable for examining the histopathological effects in the organs to simulate human T2DM [22]. Since some seaweed such as *Sargassum fusiforme* has exhibited arsenic toxicity effects [23], it is of great importance to investigate the safety or efficacy of the seaweeds on the three vital organs (liver, kidney, and pancreas) in T2DM by observing any histopathological changes. The kidney and liver play an important role in the excretion and elimination of undesirable substances from the body. The pancreas, in contrast, plays an essential role in the regulation of micronutrient metabolism. The progressive degenerations in *β*-cell function of the pancreas during the development of T2DM in humans has limited accessible information on the morphological changes of the human pancreatic islets, due to the lack of noninvasive techniques for visual observation [22]. Any systemic metabolic alterations pertaining to insulin insensitivity, insulin secretion, and loss of glycemic control are reflected by changes in the islet structure, size, or function [22]. This is particularly apparent here with the islets shrinkage (atrophy), cellular degeneration, and clear decrease in the area occupied by *β* cells, in the diabetic animals due to the combined effects of the long-term high-calorie diet and mild STZ-induced pancreatic injury (Figures 1(b)–1(g)).

Decreases in *β*-cell mass, fat deposition into the islets, and deposition of intraislet amyloid are common features of end-stage diabetes in human [22]. In contrast, the pancreas sections of the diabetic rats examined here showed alterations such as islets shrinkage (atrophy), irregular islets, cellular swelling, *β*-cell vacuolation and apoptosis, and the presence of necrotic cells (Figures 1(b)–1(g)), similar to previous findings [24]. The degeneration of the islets of Langerhans with *β*-cell loss is a significant lesion after insulin resistance [22], related to the prolonged high-calorie diet. The islet atrophy through *β*-cell loss that remains a thickened layer of peripheral cells (non-*β*) led to the progression of T2DM [25]. The extensiveness of these injuries in T2DM rats was noticeably lessened by SP extracts and metformin (Table 2). Both SP extracts significantly suppressed further damage to endocrine cells, evidenced by the decreased number of necrotic cells. This effect by SP is of important significance because cell necrosis is an irreversible process, whereas cell degeneration is reversible with the help of a good glycemic-control agent to enable it to function normally again.

*β*-cell regeneration by metformin in alloxan-induced diabetic rats have been previously reported [26] and are similarly observed here. Treatments of diabetic rats with various plants [27–30] and seaweed (*Ulva rigida*) [31] extracts have been reported to possibly cause pancreas regeneration. Beta-cell regeneration is known as one of the four means by which remedial plants demonstrate antihyperglycaemic activity [32]. However, the effect of SP may be through the prevention of *β*-cells death and recovery of the partly injured *β* cells [33]. We previously reported that SP extract supplementation to T2DM rats did not produce any significant increase in plasma insulin secretion level even after 22 days of treatment [13]. Thus, the organ protective effect and glycemic control by SP [13] is more likely due to the antioxidant action and increasing insulin sensitivity and response [32, 34]. The protective or restorative effects of SP were significantly evident in the DE300 and DW300 rats from the observed amelioration of the pancreatic cells.

The livers of the control untreated diabetic rats showed disoriented cellular structure with degenerations such as glycogen deposition (nucleus at the canter) and fatty changes (nucleus located at the peripheral cell membrane), pyknotic nuclei with acidophilic cytoplasm, similar to other experimentally induced diabetic animal models [35, 36]. The major changes in diabetic livers were hydropic swelling, disarrangement in hepatocytes, microvesicular vacuolization, granular degeneration, and necrotic cells [37]. Most of the SP extracts and metformin significantly attenuated the extent of hepatic damages. The only exception was in the DE300 group that showed severe damages which indicated the toxic effects of excessive SP ethanolic extract. Nevertheless, ethanolic extract at 150 mg/kg and water extract at 150 mg/kg significantly reversed diabetes-induced histopathological alterations in the liver. The hepatoprotective activity of *Sargassum polycystum* may possibly be due to its antioxidant pigments or sulphated polysaccharides as previously hypothesized [13, 38].

Hepatocytes in all the SP and metformin treated groups showed little or no glycogen deposition. A reduction in net synthesis of glycogen from glucose would lead to glycogen deposition in hepatocytes of diabetic rats. This could be reversed by an increase in insulin sensitivity in insulin-target tissues. Insulin-deficient diabetic animals have lower liver glycogen synthase phosphatase activity, resulting in the defective deposition of glycogen in the livers [39, 40]. Glycogen deposition is inversely correlated to glucose uptake and the severity of insulin deficiency [41]. Hence, the positive change in the glycogen content observed in the diabetic rats' livers is a good indicator for the antihyperglycemic properties of SP and metformin.

The observed hepatocytes fatty degeneration is linked to insulin deficiency and the dysregulation of mitochondrial *β*-oxidation of fatty acids. This led to the esterification of fatty acids to triglyceride in the cytoplasm, which is characterized by the presence of multiple triglyceride droplets within the hepatocytes [42]. Hepatocytes in most SP treated diabetic animals (except the DE300 group) were ameliorated from these fatty deteriorations.

Major metabolic diseases such as T2DM, obesity, and atherosclerosis are inflammatory states, and the responses to these conditions are mediated by macrophages like Kupffer cells [43]. Kupffer cells are mobile macrophages, adhering to the endothelial lining and located at periportal sinusoid. Kupffer cells are activated in response to overnutrition, whether a high-fat diet or a high-sucrose diet, which resulted in the fast development of hepatic insulin insensitivity leading to disorders in lipid metabolism [43] and hepatic insulin resistance [44]. Kupffer cells execute two roles: either (1) as a mediator of damage or (2) as a protector during the regeneration and repair processes [45]. The hepatic histological observations here showed that the severities of injuries in the SP treated rats when compared to DC rats (except in the DE300 group) were confined to cellular swelling and the presence of kupffer cells.

The hepatoprotective properties of SP in various other nondiabetic conditions were previously reported. SP ethanolic extract (125 mg/kg body weight) was hepatoprotective in hepatitis rats [46]. Acetaminophen-induced lipid peroxidation in rats pretreated with 100 and 200 mg/kg body weight of SP ethanolic and aqueous extracts showed no hepatic toxicity [47]. SP ethanolic extract (200 mg/kg body weight) also improved the hepatic mitochondrial antioxidant defence system against free radicals [38]. The SP hepatoprotective and antioxidant properties [13, 14] may partly account for the enhancement in insulin sensitivity in the diabetic rats.

Diabetic nephropathy is one of the most common complications of diabetes that is characterized by glomerular basement membrane thickening, hypertrophy and atrophy of the glomerular and tubular cells, glomerular hyperfiltration, and accumulation of extracellular matrix components in the glomerular mesangium and tubular interstitium, [48, 49] as well as the ultimate loss of renal functions. Diabetes damages renal tissues by (1) hyperglycemia and hyperlipidemia that brought about degenerations in convoluted tubules in the cortex [50] and (2) inflammatory processes [51]. The inflammatory cytokines induced by oxidative stress causes basement membrane thickening and accumulation of extracellular matrix components in the glomerular mesangium and tubular interstitium [52]. Significant losses of renal tissues occur in prolonged diabetic conditions [53]. The T2DM rats' kidneys showed acute cellular swelling and hydropic degeneration of tubules, widening of bowman's space, glomerular atrophy, congestion of capillaries, and tubular necrosis. The degree of these degenerations and necrosis decreased markedly in DE150, DW150, and DM groups. In contrast, DE300 and DW300 caused severe and moderate injuries, respectively, to the renal tissues. Although the DE300 and DW300 showed beneficial effects on the pancreas, this dose caused kidney and liver tissues injuries not shown by the lower doses of SP extracts.

The degree of proteinuria in male rats is directly correlated with hyaline droplet formation [54]. The protein in urinary filtrate forms the hyaline-like tubular cast that is distinctive in nephritic kidneys. Hyaline casts are not specific to diabetic nephropathy, but can also be observed in normal people. The observed hyaline cast protein within the tubular lumen in the DW150 rats' kidneys reflected protein reabsorption and proteinuria.

Uncontrolled hyperglycemia and hyperlipidemia are factors for diabetic nephropathy progression [55, 56], which trigger oxidative stress [57–59] and vascular oxidative stress [60]. Apoptosis also cause tubular changes and tubular atrophy in various renal diseases and diabetic nephropathy [61–63]. Antioxidants can decrease the kidney exposure to these oxidative challenges [64]. SP extracts possess potent antioxidant properties [47]. However, the DE300 rats exhibited necrotic and degenerative cells, whereby most of convoluted tubules had nuclear damage and cytoplasm loss, indicating toxicity at excessive dosage. This was verified by the cell scoring results of the renal sections. The severity of the injuries was markedly reduced in the 150 mg SP extracts/kg body weight dose on the diabetic rats.

In conclusion, this study indicated that the SP extracts at 150 mg/kg body weight were beneficial in alleviating histological injuries in diabetic animal tissues and organs. The 300 mg/kg body weight doses were beneficial to the pancreas but may be toxic to the kidney and liver of the diabetic rats.

## Figures and Tables

**Table 1:** Changes in blood glucose level (mmol/L) of rats during the treatment period (Mean ± SE).

Days	NC	DC	DE300	DE150	DW300	DW150	DM
D 1	4.9 ± 0.3^Ba^	20.3 ± 0.8^Aab^	19.8 ± 2.5^Aa^	20.7 ± 2.0^Aa^	19.2 ± 1.3^Aa^	19.3 ± 0.5^Aa^	19.4 ± 2.0^Aa^
D 7	5.4 ± 0.3^Ca^	20.8 ± 1.7^Aab^	17.3 ± 1.2^ABab^	16.5 ± 0.7^Bb^	18.1 ± 1.4^ABab^	18.3 ± 0.8^ABa^	16.1 ± 1.4^Bab^
D 13	4.9 ± 0.1^Ca^	18.3 ± 0.1^Ab^	15.5 ± 0.7^ABb^	15.4 ± 1.0^ABb^	16.6 ± 1.6^ABab^	20.1 ± 2.0^Aa^	12.4 ± 3.0^Bb^
D 19	5.4 ± 0.7^Ea^	22.4 ± 0.6^Aa^	14.6 ± 0.5^DCb^	15.6 ± 1.1^BCb^	15.9 ± 1.4^BCab^	18.0 ± 0.4^Ba^	11.9 ± 1.5^Db^
D 22	5.2 ± 0.2^Ea^	19.9 ± 1.1^Aab^	14.6 ± 0.6^BCb^	16.2 ± 1.2^BCb^	14.2 ± 1.1^Cb^	17.4 ± 1.1^ABa^	10.5 ± 1.0^Db^
HA %	+5.80% Increase	−2.00% Decrease	−35.60% Decrease	−27.80% Decrease	−35.2% Decrease	−10.92% Decrease	−84.76% Decrease

^A, B, C, D, E^Values with the same superscript/s within row do not differ significantly at *P* < 0.05. ^a,b,c^Values with the same superscript/s within column do not differ significantly at *P* < 0.05  (*n* = 6). HA: hypoglycemic activity. NC: normal control group. DC: diabetic control group. DE300: diabetic group treated with 300 mg/kg/BW of the ethanolic extract. DE150: diabetic group treated with 150 mg/kg/BW of the ethanolic extract. DW300: diabetic group treated with 300 mg/kg/BW of the water extract. DW150: diabetic group treated with 150 mg/kg/BW of the water extract. DM: diabetic group treated with 250 mg/kg/BW of metformin.

**Table 2:** Percentage of total damaged cells in the experimental animals at the end of study.

Treatments	Endocrine pancreas (islets of Langerhans)		Liver		Kidney	
	Percentage of total damage	Difference (%)	Percentage of total damage	Difference (%)	Percentage of total damage	Difference (%)
NC	4.60 ± 0.82^D^	—	5.3 ± 0.2^F^	—	3.8 ± 0.1^G^	—
DC	51.14 ± 1.57^A^	91.00%	62.8 ± 0.6^B^	91.56%	64.5 ± 0.3^B^	94.0%
DE300	29.46 ± 1.47^C^	84.38%	65.1 ± 0.6^A^	91.86%	69.4 ± 0.3^A^	94.5%
DE150	34.45 ± 2.17^B^	86.65%	5.4 ± 0.2^F^	1.85%	13.2 ± 0.4^F^	71.2%
DW300	31.55 ± 1.25^BC^	85.42%	32.9 ± 0.4^C^	83.9%	45.8 ± 0.4^C^	91.7%
DW150	35.17 ± 0.83^B^	86.92%	19.8 ± 0.5^D^	73.23%	17.4 ± 0.1^D^	78.2%
DM	30.18 ± 1.18^C^	87.76%	7.8 ± 0.4^E^	32.00%	15.2 ± 0.4^E^	75.0%

^A,B,C,D,F^Values with the same superscript/s within column do not differ significantly at *P* < 0.05. Values are expressed as Mean ± SE (*n* = 6). Difference percentage was calculated for treated groups compared to NC group. NC: normal control group. DC: diabetic control group. DE300: diabetic group treated with 300 mg/kg/BW of the ethanolic extract. DE150: diabetic group treated with 150 mg/kg/BW of the ethanolic extract. DW300: diabetic group treated with 300 mg/kg/BW of the water extract. DW150: diabetic group treated with 150 mg/kg/BW of the water extract. DM: diabetic group treated with 250 mg/kg/BW of metformin.
